# Effectiveness of Therapeutic Education in Patients with Cancer Pain: Systematic Review and Meta-Analysis

**DOI:** 10.3390/cancers15164123

**Published:** 2023-08-16

**Authors:** Ana María González-Martín, Iván Aguilera-García, Yolanda Castellote-Caballero, Yulieth Rivas-Campo, Antonio Bernal-Suárez, Agustín Aibar-Almazán

**Affiliations:** 1 Department of Education and Psychology, Faculty of Social Sciences, University of Atlántico Medio, 35017 Las Palmas de Gran Canaria, Spain; 2 Department of Psychology, Higher Education Center for Teaching and Educational Research, Plaza de San Martín 4, 28013 Madrid, Spain; 3 Department of Health Sciences, Faculty of Health Sciences, University of Jaén, 23071 Jaén, Spain; 4 Faculty of Human and Social Sciences, University of San Buenaventura-Cali, Santiago de Cali 760016, Colombia

**Keywords:** cancer, neoplasia, pain education, education in neuroscience of pain

## Abstract

**Simple Summary:**

Cancer persists as a major cause of global suffering and burden, with breast, lung and colorectal cancer leading the statistics. Cancer pain, influenced by various factors and varying in intensity, significantly affects patients, being a crucial marker related to survival and quality of life. The objective of this study is to analyze the effectiveness of pain education in those patients with pain derived from an oncological process. There is evidence that a pain education program for cancer patients can decrease mean and present pain intensity at least in the medium term, although it does not appear to affect worst reported pain.

**Abstract:**

(1) Objective: To review the existing evidence on pain education in patients with pain derived from an oncological process. (2) Methods: A systematic review was conducted using the databases Pubmed, Web of Science, PEDro, and Scopus. The selected studies had to incorporate instruction about the neurophysiology of pain into their educational program. The target population was cancer patients who had suffered pain for at least one month. The methodological quality of the articles collected was assessed using the PEDro scale. (3) Results: Some 698 studies were initially identified, of which 12 were included in this review. Four different models of pain education programs were found in the studies’ interventions. Pain intensity, pain experience, quality of life, pain tolerance, and catastrophism were the variables that appeared most frequently. (4) Conclusions: This review demonstrates that pain education in patients with cancer pain may produce effects such as decreased pain intensity and catastrophism. Knowledge about pain also seems to increase. However, no benefit was reported for patients’ overall quality of life. Therefore, more research is needed to clarify the effects of these interventions on the oncology population.

## 1. Introduction

Cancer continues to be a leading cause of morbidity and mortality, presenting a major economic problem worldwide [1]. According to the International Fund for Research on Cancer [2], breast and lung cancer were the most diagnosed in 2020, with colorectal cancer being third. In recent decades, the incidence and prevalence of cancer in developed countries have increased for various reasons, including improvements in screening and detection programs, older populations, and improved effectiveness of treatments for obesity [3,4,5]. In 2020, Santucci et al. [6] reviewed relevant studies and determined, from a global perspective, that mortality was decreasing in most common cancers except for pancreatic and lung cancers in women. Additionally, some data show that more than 80% of people with breast cancer exceed five years of life after diagnosis [7]. However, it is also true that the five-year survival rate for any type of lung cancer remains low; 17%, according to the American Society of Clinical Oncology [8]. The most frequently diagnosed cancers in Spain are colon and rectum, breast, lung, and prostate [9]. The Spanish Society of Medical Oncology estimates that in 2040, incidences in Spain will increase by almost 22% compared to 2022 [4]. In addition to all the data mentioned above, we must add the impact that COVID-19 has had on health systems and, in particular, on people with cancer. For example, in Spain [10], one in five people were either not diagnosed or diagnosed late with cancer between March and June 2020, while the number of chemotherapy treatments decreased by 9.5%. According to a report by Collateral Global [11], while we will never know the full impact COVID-19 had on cancer patients, we must investigate whether the increase in late diagnoses led to a reduction in life expectancy.

Cancer pain is a common symptom that can be caused by cellular, histological, or systemic changes underlying a neoplastic event [12]. Since cancer is a dynamic disease, data about pain can vary. Approximately 38% of all cancer patients who report pain rate it as moderate or severe [13]. Studies show prevalences of between 24 and 60% in patients having active treatment, increasing in more advanced cases (62–86%) and during the end of life [14,15]. When exploring why cancer patients in Canada and the USA seek consultation, authors concluded that pain was the most frequent reason and that approximately 30% of visits could have been avoided [16]. Importantly, the correct approach to cancer pain is connected to several key points, since pain is the independent indicator that is most directly related to survival [17,18]. Almost half of cancer patients report chronic pain as well as depressive symptoms and a low quality of life [19,20]. Pain is also frequently associated with tumor progression [21].

The management of cancer pain is carried out by a combination of pharmacological and non-pharmacological treatments. The WHO analgesic ladder [22] is the most widely used pharmacological strategy for the management of cancer pain, presenting an efficacy of approximately 80% [4,23]. The application of the WHO algorithm is complemented by the administration of adjuvants such as antidepressants or corticosteroids [24] with the aim of reducing opioid dosage and providing treatment efficacy [25]. However, the use of some non-pharmacological therapies can affect the multidimensionality of pain, making our approach more comprehensive. This, together with the considerable appearance of side effects in pharmacological strategies, makes the non-pharmacological approach an essential part of the treatment of cancer patients [26]. Education in cancer pain is a non-pharmacological intervention applicable to pain management in cancer patients, defined as the method of application or content in patients with pain, derived from an oncological process [27]. In recent decades, pain education has been increasingly introduced into educational interventions aimed at the cancer population with pain. These educational plans not only involve education about the neurophysiology of cancer pain, but also bring together a wide variety of heterogeneous interventions with different therapeutic objectives such as learning to report pain to professionals, the appropriate use of opioids, and relaxation techniques, among others [28].

Some reviewers [20,29,30] have analyzed the effectiveness of these educational interventions in cancer patients. However, these studies are not recent, so an analysis of more recent trials is needed. Similarly, in a more current systematic review that did not perform meta-analysis [31], a systematic review was conducted with the aim of seeking evidence of the effectiveness of patient education interventions in older adults with cancer, but concluded that currently available data on their effectiveness in the field of geriatric oncology are lacking, so more studies are needed to evaluate the effectiveness of these programs adapted to the specific circumstances of the elderly. Therefore, the objective of this review is to analyze the effectiveness of pain education in those patients with pain derived from an oncological process.

## 2. Materials and Methods

This systematic review seeks to evaluate the effect of therapeutic education on the neuroscience of pain in older adults diagnosed with cancer. The study was carried out following the PRISMA 2020 guidelines and the pre-established protocol registered in PROSPERO (CRD42023430108). Additionally, the methodological recommendations of the Cochrane Handbook for the Development of Systematic Reviews of Interventions were followed [32].

### 2.1. Information Sources and Search Strategy

The literature search took place in January and February 2023. The scientific databases Pubmed, Scopus, and PEDro were used to collect the trials included in this review. The parameters of the literature search were established based on the Cochrane PICOS System for recognizing patients diagnosed with cancer or neoplasia. The interventions of interest were therapeutic education about the neuroscience of pain, education about the neuroscience of pain, education about pain, and explaining pain compared to traditional cancer management. The outcome was pain.

### 2.2. Inclusion Criteria

The inclusion criteria established for this review were as follows: (i) Educational interventions must have had at least one section dedicated to explaining the neurophysiology of pain to patients, as developed by healthcare professionals; (ii) All patients diagnosed with neoplastic processes were included, whether they still suffered from the disease or were survivors of it. Additionally, all types of cancer at any stage were included; (iii) The pain suffered by patients had to be clearly associated with the neoplastic process, either as a result of treatment (chemotherapy, radiotherapy, or tumor surgery) or the natural course of cancer; (iv) The articles were about clinical trials, randomized or not; and (v) Pain or any related aspect, including intensity or pain knowledge, appeared as a measurement variable.

### 2.3. Exclusion Criteria

Studies were excluded if (1) pain education was combined with psychological therapies (hypnosis or cognitive-behavioral therapy), therapeutic exercise, or mindfulness and the results could not be analyzed in isolation; (2) the sample consisted of caregivers, family, patients not diagnosed with cancer, or health professionals were excluded; (3) variables such as depression were measured exclusively, if the reported oncological pain lasted for less than one month, if the patient’s life expectancy did not exceed three months, or if samples consisted of minors or patients unable to understand and complete questionnaires (or any educational material); (4) patients’ cognition was limited and did not include the ability to understand, speak, or read fluently, this was also a reason for exclusion; (5) people who had received invasive treatment or radiation therapy within the two weeks prior to study inclusion, (6) had major surgery within two weeks before starting the trial, (7) suffered from psychotic disorders, or (8) who were pregnant. Furthermore, articles focused on patients who (9) consumed alcohol or drugs, (10) had been the subject of an educational intervention in the past, or (11) presented metastases in the central nervous system at the time of the study were also excluded.

### 2.4. Study Selection Process

The online tool Rayyan (https://rayyan.qcri.org/welcome, accessed on 23 January 2023) was used to assist article selection. Using this tool, the articles obtained from each database were collected and duplicates were eliminated. A screening process was carried out based on the title and abstract, classifying those articles that met the established criteria as included. Two researchers were responsible for inclusion decisions, ensuring impartiality. An Agreement Percentage of 85% was estimated, which allowed calculating the percentage of agreements between reviewers in relation to the total number of decisions. In the case of discrepancies, a third author made the final decision to include or exclude the article.

### 2.5. Data Extraction

Relevant data were collected from selected articles, such as year of publication, country of origin, and authors. Additionally, participants’ characteristics, the sample size, and the distribution of the groups were extracted. Details of interventions performed in both the experimental and control groups, including the modality of the intervention, were recorded. The type of variable used and the tests used for its evaluation were identified, as well as the follow-up time and the statistical values obtained.

### 2.6. Methodological Quality Assessment

The PEDro scale was used to measure the methodological quality of the trials selected in this review. This scale was developed by a consensus of expert epidemiologists based on the Delphi list. It was adapted and translated for use in Spain in 2012 with the help of the Spanish Association of Physiotherapists and the meta-analysis unit of the University of Murcia. The tool has been widely used for 23 years within evidence-based physiotherapy. It is essential to identify both the internal validity of the trials and their statistical significance for the interpretation of results [33].

The PEDro scale is made up of 11 criteria. The first is related to external validity and is not integrated into the calculation of the final score. Meeting a criterion will add up to one point (as long as it is clearly described). Trials that achieve nine or ten points are considered methodologically excellent. Between six and eight is considered good quality, between four and five regular, while less than four is considered poor [34].

### 2.7. Analytical Decisions for Meta-Analysis

We used statistical estimators, such as mean difference (MDS) and 95% confidence interval (CI), or linear regression beta with 95% CI to synthesize the results. Heterogeneity was assessed using Cochran’s Q test (heterogeneity between included studies) and the I^2^ statistic (total variability). A subgroup analysis was performed according to the scale of measurement of intensity or pain knowledge. Additionally, publication bias was assessed using the funnel plot. All analyses were conducted using the Comprehensive Meta-Analysis V4 software.

## 3. Results

### 3.1. Selection of the Studies

**Figure 1:**
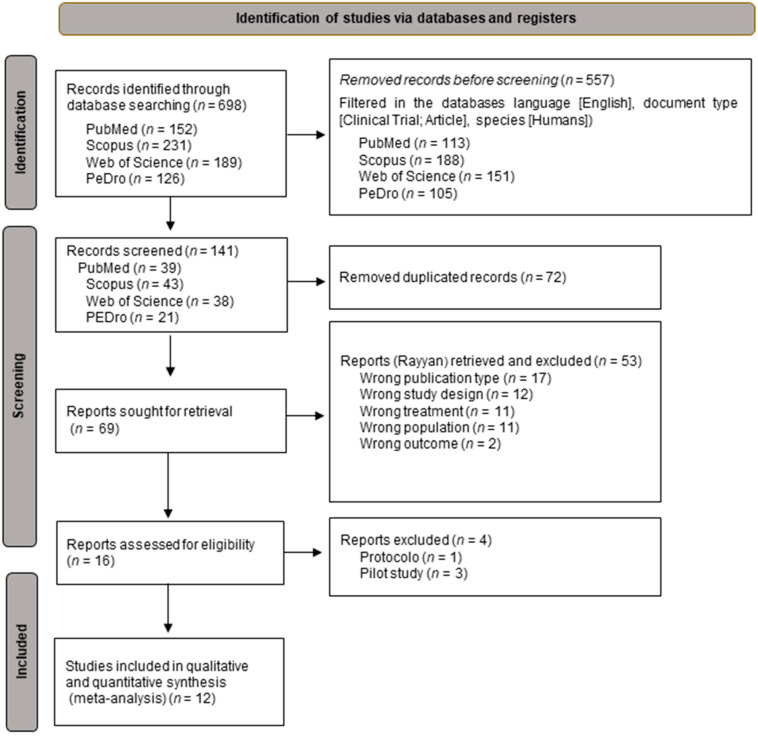
Flow diagram of the study selection process.

A search of various databases was carried out, yielding a set of 698 articles. This was reduced to 141 items using automation filters. Subsequently, 72 duplicate articles were eliminated, leaving a total of 69 that were submitted for evaluation. After completing that assessment, articles were analyzed to determine their suitability. Only 12 articles [35,36,37,38,39,40,41,42,43,44,45,46] met the established inclusion criteria (Figure 1).

### 3.2. Methodological Quality

The methodological quality of the studies was assessed using the PEDro method. Assessments were obtained directly from the PEDro website studies for seven studies [35,36,37,38,44,45,46], while the others [40,41,42,43,44] were evaluated manually. Five of the included articles were classified as ‘good’ in terms of methodological quality [39,41,42,43,46] and seven received a rating of ‘fair’ [35,36,37,38,39,44,45] (Table 1).

### 3.3. Characteristics of the Studies

The articles included in this systematic review were experimental studies published in English [35,36,37,38,39,40,41,42,43,44,45,46] between 1997 and 2023. Publications were identified in different countries, such as the Netherlands [35,36,37,38,45], Turkey [39], Taiwan [40], Belgium [41], Germany [42], the United States [44], and Kenya [43]. In total, 709 people linked to the experimental group participated. The sample size in the 12 articles included in this systematic review ranged from 29 [42] to 313 individuals [35,36].

Most interventions lasted eight weeks [35,36,37,38,39,40,41,44]. Two studies had no control group [41,42], while the others used standard cancer care as a comparison [35,36,37,39,40,44,46]. The interventions had a minimum duration of one week [35,36,37,38,39,43] and a maximum of six months [44]. Table 2 provides full details of the articles included in this review.

### 3.4. Study Results

Ten of the studies included in this review supported the favorable results of the educational intervention for pain management. Wit et al. [35] and de Wit et al. [36] found that patients with nursing district were mostly elderly (*p* < 0.001) and women (*p* < 0.05) compared to those without a nursing district. Significant differences were also observed in the employment (*p* < 0.05) and civil (*p* < 0.05) situations between the groups. Likewise, Yildirim et al. [39] and Lai et al. [40] demonstrated that structured pain education effectively improved the experience of pain in hospitalized cancer patients and suggested its implementation in clinical practice (F04.01 *p* = 0.00).

Studies by Oldenmenger et al. [37,38] supported the effectiveness of the combination of cancer care, pain education programs, and specialized consultations in the experimental group. Pas et al. [41] observed a significant decrease in pain intensity (*p* = 0.001) compared to baseline conditions. Kim et al. [43] found a significant decrease in pain intensity at week, including the worst pain (*p* < 0.01). Van der Peet et al. [45] showed significant differences in pain intensity between the intervention and control groups at different points in the study (*p* = 0.00) in patients who initially had a score of 7–10. Yates et al. [46] found that participants in the intervention group experienced a significantly greater increase in knowledge about pain and an improvement in pain intensity. These findings may be relevant for understanding the impact of cancer care combined with pain education programs on patients’ daily lives.

Looking at other studies, significant changes were found within groups, but no major differences were evident compared to the control groups. In the study by Wells et al. [44], no influence on long-term pain outcomes was observed following continuous access to pain information, either initiated by the patient or the health care provider. In contrast, De Groef et al. [42] found significant differences in pain measured by the McGill Pain Index (PDI), but no significant differences using the Numerical Pain Scale (NRS) (*p* = 0.49).

#### 3.4.1. Medical Information and Type of Cancer

Medical data related to the neoplastic condition of the participants were reviewed. In the two studies by Wit et al. [35,36], it was observed that in the control group without a nursing district, 35% had breast tumors, 11.7% had tumors in the genitourinary organs, and 15.5% had cancer of digestive organs and peritoneum. Some 62% had metastases, and 18.4% had local spread of cancer. In the experimental group, 24.5% had genitourinary organ cancer and 23.6% had breast cancer, while 19.8% had bone, connective tissue, or skin cancer. A total of 51.9% had metastases and 23.6% showed regional spread of cancer. In the control group with a nursing district, 37.3% had genitourinary cancer, 29.4% had breast cancer, and 15.7% were classified in the category of others.

Oldenmenger et al. [37,38] found that in the control group, 29.7% had breast cancer, 27% had gastrointestinal cancer, and 16.2% had urogenital cancer. Some 86.5% had metastases and the rest had a local spread of cancer. In the experimental group, 37.1% had breast cancer while 77.1% had gastrointestinal and urogenital tumors. A total of 77.1% had metastases and in 7%, the cancer was localized.

Yildirim et al. [39] reported that in the sample, 40% had cancer of the digestive organs and peritoneum, and one in four had neoplasms in genitourinary organs. Some 45% had a disease duration of between one and three years, and 30% had less than one year. A total of 60% had metastases and the rest had a localized neoplasm. Some 80% of patients received chemotherapy. In the experimental group, 45% had tumors in the digestive organs and peritoneum, and one in five had breast cancer. For 40% of the patients, the duration of the neoplasm was less than one year, and for 35% it was one to three years. Some 65% received chemotherapy. Likewise, Lai et al. [40] described that all patients in the control group had metastases, 46.7% of them stage IV and 40% stage II. In the experimental group, 26.7% had stage IV, and eight of the 15 were given chemotherapy that month. No notable differences between groups were observed with respect to the oncological theme in any of the studies.

#### 3.4.2. Painful Experience

Wit et al. [35] and Yildirim et al. [39] addressed information on the affectation in the different dimensions of pain. Both sets of authors [35,39] used the McGill Pain Questionnaire as a tool to evaluate the individual pain experience of each patient at the beginning of the study and at two weeks, measuring the function of three components: sensory, affective, and evaluative. Wit et al. [35] found that the differences between groups at baseline are not relevant. For their part, Yildirim et al. [37] reported that the score in the dimensions of the control and experimental groups did not express statistically significant changes either at the beginning of the study or in the measurement at two weeks (*p* > 0.05).

#### 3.4.3. Pain Intensity

In the search to learn whether pain education in cancer patients can reduce pain, the studies included in this review collected information about participants’ pain with different instruments. One of the tests used was the Numeric Rating Score (NRS) in which participants indicated from 0 to 10 the intensity of perceived pain. This was employed by Wit et al. [35], Yildirim et al. [39], and De Groef et al. 2023 [42].

Wit et al. [35] collected information at the beginning of the study and at two weeks after application as well as at the fourth, and eighth weeks. No differences were found at baseline between the groups with respect to either of the two parameters (for example, the intensity of pain being 3.3 with a SD (standard deviation of 2.3). At four weeks, the value of both control groups remained the same, while the experimental groups scored ≈ 2.3 P. Likewise, Yildirim et al. [39] analyzed this parameter at the beginning of the study and at the second, fourth, and eighth weeks of the intervention. The group that received the pain education started with a present pain of 3.1, a worst pain of 4.6, and 1.25 as the last reported pain. In the eighth week, these values were 1.20, 3.75, and 0.65, respectively. The group that received the educational program decreased significantly in the parameters of present pain and last reported pain. Unlike in previous studies, De Groef et al. 2023 [42] reported no significant differences with the NRS (mean difference of 0.3 and *p* = 0.49).

The most frequent instrument for assessing the levels of intensity of mean, current and worse pain of cancer patients was the Brief Pain Inventory (BPI), which was used in seven of the articles [37,38,40,43,44,45,46].

Oldenmenger et al. [36] reported that in the control group, the average pain was 5.7, the worst pain was 7.9 points, and the current perceived pain was 4.2. The experimental group had a mean pain of 6.2, a worst pain of 8.1, and a current pain of 5. At the eighth week of measurement, the data from the experimental group reflected a mean pain of ≈3.7 and a current pain of ≈2.2. Depending on the other group, the data referred to in the average are ≈4.3 and in the currently reported ≈2.9. Likewise, Oldenmenger et al. [38] highlighted that the mean pain intensity variable of the control group was 1.13, compared to 1.95 for the experimental group (*p* = 0.03). Regarding the current pain variable, the control group stood at 0.67, while the experimental group was at 1.50 (*p* = 0.016). In contrast, there were no significant differences between the worst pain reported between the two groups (1.16 in the control and 1.28 in the experimental).

Lai et al. [40] found that after the five-day intervention, the control group presented a 5.53 intensity in the worst perceived pain, 2.47 for the last pain, 3.73 for medium intensity, and 3.47 for the present pain. In the experimental group, the variables were different: 5.33 for the worst pain reported, 0.93 for the last pain, 2.80 for the mean intensity of pain, and 1.73 points for the present pain. The starting values do not present relevant differences. Comparing the control and experimental groups once the intervention was completed, significant improvements were observed in the mean and current pain intensities. Similarly, Kim et al. [43] observed a significant improvement in pain intensity in one week, both in the worst pain (from 7.3 to 5.7, *p* < 0.01) and the mean pain (from 4.6 to 3.8, *p* < 0.01). Van der Peet et al. [45] demonstrated that at four weeks, a decrease in pain level was observed, although it was not maintained at eight weeks and only those patients with a high baseline pain score experienced significant decreases in pain. T0 = 4.71 (2.21) Q1 (week 4) 3.78 (2.63) *p* = 0.001, Q2 (week 8) 4.00 (2.17) *p* = 0.14. Finally, Yates et al. [44] found that after one week of the intervention, the following BIS values were reported: T0 = 4.1 (1.8) T1 = 3.9 (1.8) T2 = 3.5 (1.7). Additionally, patients in the intervention group showed a greater reduction in willingness to tolerate pain and concerns about addiction and side effects, as well as tolerance to pain-relieving medications.

The VAS (Visual Analogue Scale) tool was only used in the study by Pas et al. [41], where the score before the application was 47 and after intervention with telemonitoring was 40. A significant increase appeared (*p* < 0.001).

#### 3.4.4. Knowledge about Pain

Understanding that pain education is based on the instruction and reconceptualization of pain in cancer patients, it is important to have a tool that allows us to assess whether the patient has understood the complexity of the painful experience. Two clinical trials focused on its assessment, measuring knowledge at baseline and two weeks after implementation of the educational program [35,37]. The instrument used in both studies was the Dutch version of the Pain Knowledge Questionnaire (PKQ-DLV). This questionnaire consists of eight pain-related questions, and the final score can range from 0 to 100 (the closer to 100, the greater the knowledge about pain demonstrated).

Wit et al. [35] put the total score, combining all four subgroups, at 54.8 points at the beginning of the study. Although the results were not reported for each of the subgroups, the authors stated that the highest level of knowledge was shown in the items ‘the use of non-pharmacological treatment relieves pain’, ‘belief that the health professional provides you with the need to go to consultation to change the medication’ and ‘pain can be relieved’. No significant differences between subgroups were observed in the initiation of the trials. In contrast, the authors realized that those with a higher level of education scored better in some items such as ‘medication should be used only when the pain is severe’ (*p* < 0.01), and ‘becoming addicted’ (*p* < 0.001) if the results are compared with patients with a lower index of education. The items in which the improvement appears in both experimental groups (district and not) are ‘taking as little medicine as possible’, ‘using a medication routine instead of one-time dose’, and ‘becoming addicted’. Similarly, Oldenmenger et al. [37] established that the control group had a pain knowledge score of 65 points while the experimental group had a score of 62. Two weeks after the application of the educational intervention, there was a significant improvement (*p* = 0.002) between the experimental group (71 points) and the group that underwent only standard oncological care (64 points).

### 3.5. Meta-Analysis

**Figure 2:**
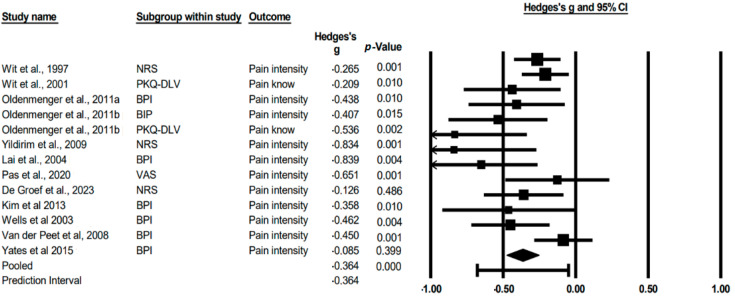
Random-effects model for analysis of change in pain from pain education in cancer patients. The black box represents the point estimate for the respective study, while the size of the box represents the population size and the horizontal line is the 95% confidence interval. The diamond-shaped figure represents the estimated point of the average effect size [35,36,38,39,40,41,42,43,44,45,46].

We were able to integrate the 11 articles into a meta-analysis to synthesize the findings. The mean effect size is −0.364, indicating a decrease in the mean effect compared to the baseline. The 95% confidence interval (−0.474 to −0.253) shows the range in which the true size of the effect in the population is expected to be. The value of Q is 22.760 with 12 degrees of freedom and *p* = 0.030. Using an alpha criterion of 0.100, we can reject the null hypothesis that the true effect size is the same in all studies, implying that there is variability in results between different studies (Figure 2).

The I square tells us that approximately 47% of the variability in the observed effects is due to the variability in the true effects and not to sampling error. The value of squared Tau, which represents the variance of the actual effect sizes, is 0.017 in g units. On the other hand, Tau, which indicates the standard deviation of the actual effect sizes, is 0.131 in g units. As for the prediction interval, if we assume that the actual effects follow a normal distribution in g-units, we can estimate that the prediction interval is between −0.679 and −0.049. This means that the actual effect size in 95% of all comparable populations falls within this range.

### 3.6. Subgroup Analysis

**Figure 3:**
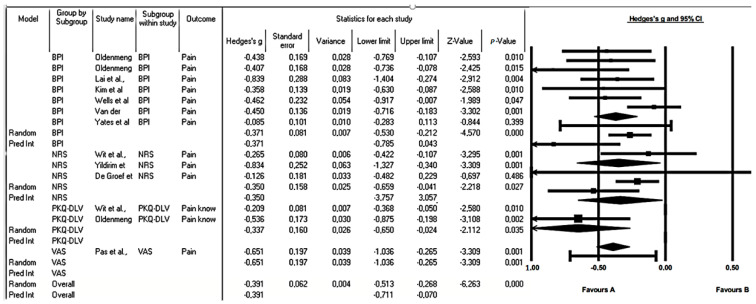
Subgroup analysis by pain assessment scales to assess the effect of education in cancer patients. The black box represents the point estimate for the respective study, while the size of the box represents the population size and the horizontal line is the 95% confidence interval. The diamond-shaped figure represents the estimated point of the average effect size [35,36,37,38,39,40,41,42,43,44,45,46].

Given the conditions of heterogeneity, a subgroup analysis was performed according to the scale used. Significant changes were evident in each subgroup (*p* < 0.05). The analysis of pain intensity from the NRS focused only on the intensity of pain represented in the magnitude of pain and at the exact moment of measurement and showed a small but significant mean effect size of g = −0.35 (*p* = 0.27). The BPI addressed not only the intensity of pain but also other aspects such as the impact of pain on daily function and the perception of pain over time. This instrument yielded a g of Hedge = 0.371 (*p* = 0.00), evidencing a small effect size but with significance (*p* = 0.000). Likewise, the article analyzed with the VAS instrument showed a decrease in pain intensity with a moderate effect size g of Hedge = 0.651 (*p* = 0.001). In contrast, the articles that focus their analysis of pain knowledge with PKQ-DLV showed an improvement compared to the control groups g of Hedge = −0.337 (*p* = 0.035) with a small effect size. Finally, the general model g of Hedge = 0.391 maintained a small but significant effect that evidences the changes with the intervention (*p* = 0.000) (Figure 3).

### 3.7. Publication Bias

The analysis was carried out using a funnel plot that included all the articles of the meta-analysis. This analysis revealed the presence of an expected publication bias, as there were articles showing different results in terms of mean difference. However, when a subgroup analysis was performed based on the assessment instrument used, it was observed that heterogeneity decreased and a more symmetrical distribution of results was shown.

## 4. Discussion

The objective of this systematic review and meta-analysis was to analyze the effect of pain education in patients with pain related to an oncological process. Although several authors have reviewed the effectiveness of educational interventions for the management of cancer pain, none have focused on measuring the effectiveness of an education program that integrates a reconceptualization of pain and an understanding of the biological processes underlying the painful experience [2,47,48,49] as this one intends. There is increasing scientific evidence about therapeutic pain education in patients with cancer pain [2]. However, despite the results of the reviewed studies regarding patient benefits, extrapolation of the results to clinical practice and interpretation remains limited. A recurring aspect in these reviews is the application of pain education within an educational plan in which different strategies appear, such as learning about analgesic pharmacology and its adverse effects or active coping strategies such as relaxation. Thus, the effectiveness of this intervention is biased by its combination with other educational strategies. Additionally, interventions were frequently heterogeneous [2,50] due to a lack of standardized protocols and poor integration of educational interventions in oncological pain management guidelines [50].

The application of pain education appears in the literature via multiple strategies such as informative videos, theoretical classes, or phone calls as well as online sessions in which the patient autonomously learns the neurobiology of pain without the help of a professional [51]. As has been reported, the exclusive use of the online format may have limitations if the patient misunderstands concepts or needs to ask for information [52]. However, written material with theoretical content could be a tool of great value for patients to review and store concepts [53]. Similarly, there is no consensus on the number and duration of pain education sessions that are useful for cancer patients, since the interventions in the studies selected in this systematic review and meta-analysis lasted a minimum of one week [35,36,37,38,39,43] and a maximum of six months [44]. Likewise, the duration of the sessions varied between 10 and 45 min. The lack of consensus regarding the optimal number, duration, and content of pain education sessions in cancer patients reflects an inherent complexity of medical care and the individualized nature of patients’ needs [54]. This lack of consensus can be attributed to several reasons such as changes in the disease and treatment, the complexity of the pain experience, and the scarcity of resources and tools in education [55], and it is important to address this issue to improve the care and well-being of cancer patients. Although this intervention has a solid theoretical framework for the treatment of central sensitization syndromes and musculoskeletal pain [53,56], it is completely different in the case of cancer pain.

To optimize the management of cancer pain, it must be taken into account that this is a staggered process that is exposed to changes [57]. Since the cancer patient communicates their interpretation of the painful experience until the pharmacological balance is reached and the resulting adverse effects are minimized as much as possible, there are processes of advice and reevaluations in which the health system may not give the appropriate response [1]. In addition, cancer pain is not a static condition but is dynamic, since the physiology that surrounds each type of cancer is different and each stage varies depending on the disease [50].

Although the educational intervention used was the same, heterogeneity in the variables measured was a feature in the reviewed studies. However, confluences appear in some variables—such as sociodemographic variables and certain population characteristics—were a constant in all reviewed studies, meaning these can be commented on due to their greater presence. These variables include gender, age, marital status, ethnicity, employment status, and religious beliefs. Regarding the total sample of trials in this review, it appears that the mean age of participants was between 50 and 61 years. More than half were women and most were married. Additionally, patients’ educational levels were collected. The measurement of this parameter became relevant when our intervention aimed to integrate ideas and reconceptualize concepts in the cancer patient. In their multiple regression analysis, De Wit et al. [36] showed that those patients who had a lower level of education and were widowed benefited more from PEP. In contrast, Lai et al. [40] suggested that a high level of education is not necessary to benefit from pain education, although they point out that more research is needed to clarify this possible relationship.

In the total sample of participants who made up the trials of Wit et al. [35,36], it was extracted that patients who had a nursing district were more frequently women and older, differed in employment and marital status, and expressed that the most significant differences in patients with a nursing district was in the location of the tumor and the anticancer therapy applied. In contrast, in the other articles that analyzed this variable, no statistically significant details were denoted. Regarding the medical information and characteristics related to the health of the cancer patients, both the clinical history and the knowledge of the health professionals who attended to the patients were sources of this information, which included the type of primary tumor, its treatment, stage, or level of dissemination. The most frequently reported tumor was breast, followed by tumors in the digestive/gastrointestinal organs. Moreover, of the 30 participants from Lai et al.’s trial [40], all had metastases. Regarding the characteristics of pain and its location, pain in the lumbosacral region and/or in the abdominal area were the most frequently reported.

The mean persistence of pain was established in a range of between 5.7 and 21 months, which makes us think that they had chronic pain and that most participants were under analgesic pharmacological treatment to alleviate their pain. Thus, it is necessary to record the type of analgesics and the dose administered to know the possible alteration in the effect of education on pain. Additionally, caution must be exercised in the interpretation of this pain since changes in the nervous system do not always perpetuate these episodes of pain, despite some evidence in this regard [57,58].

Learning whether pain education is effective for reducing pain intensity was an objective of five of the trials in this review. There was evidence of a decrease in the variables of mean, present, or last reported pain intensity in the trials of Wit et al. [35], Oldenmenger et al. [37], and Yildirim et al. [39]. These authors also stated that this effect did not occur in the intensity parameter of the worst pain reported. The concordance of these results could be due to the application of a similar pain education program (PEP), as well as the agreement in measurement points. However, Lai et al. [40] do not agree on measurement points being short-term. Despite this, significant improvements in the current intensity and mean of pain were found. In contrast, these effects are not reported in the worst reported pain variable and the last perceived variable [40]. Consistent with the results of these trials, it appears that pain education could have short-term effects on the variables of mean and/or present pain intensity. However, when the follow-up of the variable pain intensity reaches three months, there may also be a significant decrease [41].

The painful experiences of cancer patients were measured in two of the reviewed articles [35,39] with the McGill Pain Questionnaire. However, in one [35], only their results collected at T0 were detailed, while in the other [39], it was indicated that there were no significant changes between groups at T1. Measurement of pain multidimensionality may not yield significant results if measured so early (at two weeks after the educational intervention). Among other things, we must bear in mind that, as health professionals, we cannot directly influence all aspects that concern pain and that this tool analyzes, which causes the patient to take an active role in pain improvement. In any case, the data regarding the painful experience were not presented transparently.

Both quality of life and interference with the daily life of cancer patients were collected in five of the articles, which measured this variable using EORTC QLC-C30 (+3) [35], BPI [37,40], SF-36 [41], and McGill Quality of Life Questionnaire (MQoL) [42]. One study indicated benefits in the areas of ‘less pain’ and ‘fatigue’ for the experimental groups [35], indicating that an improvement in fatigue could be related to perceived pain intensity. Similarly, two studies [38,40] reported a significant difference in the overall score of the experimental group compared to the control. Lai et al. [40] only reported a significant improvement for the five-day intervention group in the general activity domain. This could indicate that, for there to be a total reportable benefit, the follow-up must be at least eight weeks. However, another study [41] reported statistically significant differences in just two weeks, but did not establish a comparison with a control group.

Two of the reviewed articles [35,37] analyzed patients’ knowledge of pain and found that all participants increased their knowledge regardless of whether or not they received the educational program, although the experimental group significantly increased their total score for this parameter compared to the control group. These two characteristics, together with the application of the same intervention design (PEP), could justify the concordance of these results. A patient’s knowledge of pain could lead to short-term benefits, although no information was collected in this review in relation to the increase in knowledge when the follow-up was greater than two weeks.

In contrast, two articles [40,42] measured pain tolerance, one of which [40] showed that cancer patients who underwent surgery had a lower pain tolerance. The fact that the experimental group presented an acceptable intensity of lower pain could indicate that when an oncological patient is instructed in their pain and approach, the acceptance of having to suffer intense episodes of pain is reduced. However, De Groef et al. [42] reported no relevant changes, while in the study by Liu et al. [59], a significant difference appeared between the two groups in the eighth week of the intervention. Finally, another variable studied in some reviewed studies was the level of participant catastrophism [35,40,42]. All three articles showed statistically lower catastrophism in the experimental group at the end of the intervention. It should be noted these three studies did not include a control group, so we cannot know if this improvement occurs without an educational program.

This review has certain limitations that should be noted for future research. The observed heterogeneity among the selected articles. Although common measurements appear, such as pain intensity or level of disease knowledge, these present a wide difference. The bulk of the longer measurements of these studies do not exceed the follow-up of eight weeks, so the effectiveness of PD can only be given for the medium term. There are no exhaustive descriptions of the content of NDT interventions in the studies, but the method and time spent. Similarly, the study with the highest quality on the PEDro Scale was five out of ten.

## 5. Conclusions

There is evidence that a pain education program for cancer patients can decrease the intensity of medium and present pain at least in the medium term, although it does not seem to affect the worst reported pain. Additionally, it is unclear whether educational interventions improve the overall quality of life in this population, but they could have a positive effect on some variables. Knowledge about pain after an education program may improve even two weeks after application. Some evidence suggests that catastrophism and/or acceptable pain intensity or tolerance decrease after a pain education program in cancer patients. With the current evidence, it would be difficult to establish a standard and evidenced method of application of pain education for this population. Therefore, more research is needed to demonstrate the effects of this intervention in the short, medium, and long term.

## Figures and Tables

**Table 1:** Methodological quality of the articles included.

Article	1	2	3	4	5	6	7	8	9	10	11	Total Score on PEDro Scale
Wit et al., 1997 [35]	Y	Y	N	Y	N	N	N	N	N	Y	Y	4
Wit et al., 2001 [36]	Y	Y	N	Y	N	N	N	Y	N	Y	Y	5
Oldenmenger et al., 2011a [37]	Y	Y	N	N	N	N	N	N	N	Y	Y	3
Oldenmenger et al., 2011b [38]	Y	Y	N	Y	N	N	N	Y	N	Y	N	4
Yildirim et al., 2009 [39]	Y	Y	Y	Y	N	N	Y	Y	Y	Y	Y	8
Lai et al., 2004 [40]	Y	Y	N	Y	N	N	N	Y	N	Y	Y	5
Pas et al., 2020 [41]	Y	Y	N	Y	Y	Y	N	Y	N	Y	Y	7
De Groef et al., 2023 [42]	Y	Y	Y	Y	N	N	N	Y	Y	Y	Y	7
Kim et al., 2013 [43]	Y	Y	N	Y	Y	Y	N	Y	Y	Y	Y	8
Wells et al., 2003 [44]	Y	Y	N	Y	N	N	N	N	Y	Y	Y	5
Van der Peet et al., 2008 [45]	Y	Y	N	Y	N	N	N	Y	N	Y	Y	5
Yates et al., 2015 [46]	Y	Y	N	Y	N	N	Y	Y	Y	Y	Y	7

Items: 1 = eligibility criteria; 2 = random allocation; 3 = concealed allocation; 4 = baseline comparability; 5 = blind subjects; 6 = blind therapists; 7 = blind assessors; 8 = adequate follow-up; 9 = intention-to-treat analysis; 10 = between-group comparisons; 11 = point estimates and variability. Y = Yes; N = No. The total score on the PEDro scale represents the sum of the points obtained in the different items of the scale (2 to 11). Each item evaluates a specific aspect of the methodological quality of the study, such as randomization, allocation concealment, blinding of participants and assessors, intention-to-treat analysis, among others.

**Table 2:** Characteristics of the included studies.

				Control Group				Experimental Group					Variables			
Author and Year	Country	Sample Size	Age Mean (SD)	N	Relevant Characteristics	Intervention	Sessions/Method	N	Relevant Character-istics	Intervention	Sessions/Method	Time	Outcome	Measuring Instrument	Assess-Ments	Results
Wit et al., 1997 [35]	Netherlands	313	15.5	154	With nursing district: 51 No nursing district: 103 65.6% women Mean age 55.9 Breast and genitourinary cancer as the most frequent tumors.	Standard cancer care	-	159	With nursing district: 53 No nursing district: 106 59.7% women Mean age 57 Breast and genito-urinary cancer as the most frequent tumors.	Standard cancer care + PEP	Initial instruction 30–60 min. Telephone sessions. 5–15 min, theoretical brochure, and lectures recorded in audio as reinforce-ment.	1 week	Painful experience	MPQ-DLV	At baseline [T0] and 2 weeks [T1].	Although all patients’ pain intensity scores decreased after discharge, scores in the intervention group decreased significantly more than for patients in the control group at follow-up (*p* < 0.01). Mean pain intensity T0 = 58 vs. T3 = 16.9 *p* = 0.01
													Medium and present pain intensity	NRS 0–10	At baseline [T0], 2 weeks [T1], 4 weeks [T2], and eighth [T3].	
													Quality of life	EORTC QLC-C30 (+3)	At baseline [T0] and at 4 weeks [T2].	
													Pain knowledge	PKQ-DLV	At baseline [T0] and 2 weeks [T1].	
Wit et al., 2001 [36]	Netherlands	313	15.5	154	With nursing district: 51 No nursing district: 103 65.6% women Mean age 55.9 Breast and genitourinary cancer was the most frequent tumors.	Standard cancer care	-	159	With nursing district: 53 No nursing district: 106 59.7% women Mean age 57 Breast and genito-urinary cancer was the most frequent tumors.	Standard cancer care + PEP		1 week	Pain intensity present/mean pain intensity	AMPI	At baseline [T0], 2 weeks [T1], 4 weeks [T2], and eighth [T3].	AMPI R^2^ 60% Short term BETA −0.47 < 0.001 Long term R^2^ 56% beta 0.72 < 0.001 Pain knowledge beta 0.12 < 0.01 Pain impact beta 0.11 < 0.05
													Worst pain intensity	(MPQ-DLV)		
													Tolerable intensity of pain	(PKQ-DLV)		
													Experience of pain	List of cancer patients		
													Pain Knowledge	(PCL-C)		
Olden-menger et al., 2011 [37]	Netherlands	72	59 (11)	37	62% women Mean age 61 Breast and gastroin-testinal cancer was the most frequent tumors. All have nociceptive pain.	Standard cancer care	-	35	69% women Average age 56 Breast cancer was the most common tumor. All have nociceptive pain. Tumor status: Locally advanced 14 (19%) Metastatic 58 (81%) Pain duration (months; median, IQR) 5 (3–14)	Standard cancer care + pain consultation + PEP	Previous PEP + Compre-hensive face-to-face assessment at the beginning of treatment.	1 week	Pain intensity	BPI	At the start of the study [T0], after two weeks [T1], after 4 weeks [T2] and after 8 weeks [T3].	Mean score for intensidad media aceptable del dolor. CG T0 = 4.6 (standard deviation (SD) = 1.7), T3 = 5.0 (SD = 1.9) IG acceptable pain decreased significantly T0 = 4.6 (SD = 1.7) T = 3 3.8 (SD = 1.7; *p* < 0.01). Difference between the groups was significant at 8 weeks (*p* < 0.05).
													Use of analgesics	OMS analgesic ladder + MEDD (table of equiv-alences		
													Need for analgesic prescription	ATC + PRN		
													Adequacy of analgesic prescription	PMI		
													Analgesic adherence	MEMS	Measured in each of the 8 weeks.	
													Knowledge about pain	PKQ-DLV	At baseline [T0] and at 2 weeks [T1].	
Olden-menger et al., 2011 [38]	Netherlands	72	59 (11)	37	62% women Mean age 61 Breast and gastro-intestinal cancer was the most frequent tumors. All have nociceptive pain.	Standard cancer care	-	35	69% women Average age 56 Breast cancer was the most common tumor. All have nociceptive pain. Tumor status: Locally advanced 14 (19%) Metastatic 58 (81%) Pain duration (months; median, IQR) 5 (3–14)	Standard cancer care + Pain consultation + PEP	Previous PEP + Compre-hensive face-to-face assessment at the beginning of treatment.	1 week	Acceptable intensity of pain Knowledge of pain	BPI + Question (NRS)	Measured at 2 weeks [T1], 4 weeks [T2], and 8 weeks [T3].	For mean pain intensity, the mean reduction was 1.13 for CG and 1.95 for GI (20% vs. 31%; *p* = 0.03). For current pain intensity, the mean reduction was 0.67 for CS and 1.50 for PC-PEP (16% vs. 30%; *p* = 0.016). At week 2, the level of pain awareness (0 to 100) was significantly better after random GI assignment (71 ± 13).
Yildirim et al., 2009 [39]	Turkey	40	20	20	75% < 60 years 55% men Tumors of digestive organs and peritoneum was the most common.	Standard cancer care	-	20	95% < 60 years old 55% men Tumors of digestive organs and peritoneum was the most common.	Standard cancer care + PEP (modified)	Initial instruction 30–40 min. Sessions of 5–15 min and theoretical brochure as reinforce-ment.	1 week	Pain intensity (present, worst, and last)	NRS	At the beginning of the study and weeks 2, 4, and 8 of the application.	The effect of the PEP on patients’ pain intensity (present, worst, and least pain intensities) and satisfaction with pain treatment was assessed at baseline 3.1 (±1.55) and after 2 weeks (mean 1.10 ± 0.85), 4 weeks (1.20 ± 1.02), and 8 weeks (1.20 ± 1.06).
													Painful experience	MPQ	At the beginning of the study and 2 weeks after application.	
													Patient barriers in relation to oncological pain management	BQ-r		
													Patient fitness	KPS	Measured at baseline.	
Lai et al., 2004 [40]	Taiwan	30	53.5	15	60% women Mean age 56 Half are undergoing chemotherapy and all have metastases.	Standard cancer care	The control group was visited by the researchers once a day for 10–15 min. However, researchers did not provide the group with any information regarding concepts of pain.	15	53% women Mean age 51.7 Half are undergoing chemo-therapy and all have metastases.	Standard cancer care + PEP	5 days with individual talk of 10–15 min. Explain a theoretical brochure and reinforce-ment of concepts.	5 days	Mean, current and last pain intensity	BPI-T brief pain inventory	Measured at the beginning of the study and the end of the intervention (5 days).	BPIT CG = pre = 4.33 ± 2.88 pos = 3.73 ± 1.83 T = 0.98 IG pre = 5.00 ± 1.07 pos = 2.80 ± 1.61 t = 2.40 Between groups = t = 0.94 F = 4.01
													Interference with daily life			
													Pain tolerance	POAB-CA		
													Catastro-phism	CSQ-Cat		
													Beliefs about sense of pain control			
Pas et al., 2020 [41]	Belgium	Not Compared To Control Group						30	Cancer survivors. 24 women and 6 men. Breast cancer was the most common tumor.	Pain neuroscience education program	Individual talk of at least 30–45 min. Theoretical pamphlet as reinforce-ment.	2 weeks	Pain intensity	VAS	Measured at baseline and 2 weeks after application.	Levels of the VAS were significantly higher pre = mean 47 (RIQ 43–65) compared to post = 40 (RIQ 34–55) mean difference = (*p* = 0.001, r = −0.44).
													Catastrophist pain	PCS		
													Quality of life	SF-36		
De Groef et al., 2023 [42]	Germany	Not Compared To Control Group						29	Mean age 50.8. All participants were breast cancer survivors. 90% with unilateral breast involvement.	eHealth intervention: personalized pain neuroscience education program for each breast cancer survivor.	22 individual sessions with graphic support material.	6 weeks	Pain-related functionality	PDI	Measure-ments at the beginning of the study (T0), post-intervention (T1), and at 3 months (T2).	1. Pain-related functioning (PDI) Pre = 27.6 (13.0) Post 1 = 22.6 (±12.3) T2 = 21.2 (±14.0) *p* = 0.0038
													Aggravating pain	NRS 0–10		
													Self-reported symptoms of hyperalgesia and allodynia	CSI		
													Physical function	PROMIS-PF-SF		
													Pain concern	PCS		
													Depression, anxiety, and stress.	DASS-21		
													Symptoms	BTMS		
													Quality of life	0–10		
													Health related to quality of life	MqoL		
													Self-efficacy	PSEQ		
Kim et al., 2013 [43]	Korea	108	59.5	54	38 (70.4) men 16 (29.6) women	General education	Pain management nurse trained each patient and caregiver together for approxi-mately 30 min.	54	35 (64.8) men 19 (35.2) women	Pain education plus telemonitoring.	Tele-monitoring regarding pain was performed by an NP every day for 1 week. The NP phoned patients and asked for their mean VAS pain score and worst VAS pain score in the last 24 h.	1 week from the first visit	Pain intensity	BPI	T0 = Baseline T1 = 1 week T2 = 2 months	Pain intensity was significantly improved at 1 week, including worst pain (7.3 to 5.7, *p* < 0.01) and average pain (4.6 to 3.8, *p* < 0.01).
Wells et al., 2003 [44]	USA	64	53.11	56	54% men and 70% metastases.	Standard cancer care	-	47.95	76 men in hotlines. 68 men in weekly call.	Weekly call.	All patients and their caregivers participated in the pain education program. Included structured and tailored components. The structured component was a 15-min videotape (‘Taking Charge of Your Pain’), side effect management, and discussion of the patient’s present pain regimen. This education program took 20 to 30 min.	6 months	Pain intensity	BPI	T0 = baseline T2 to T6 monthly for 6 months	Average pain (0–10) t0 = 4.18 (2.10) T1 = CG 4.50 (2.17) IG1 4.00 (2.05) IG2 3.97 (2.12) F = 0.44 *p* = 0.05 Continuous access to pain-related information using a patient- or provider-initiated format did not affect long-term pain outcomes.
Van der Peet et al., 2008 [45]	Netherlands	120	61	62	Mean age 60 Men = 38.3%	Standard cancer care	-	58	Mean age 62 Men = 52.1%	Skilled nursing care at home, at the same time as the care received from their doctor, including PEP and follow-up of symptoms other than pain. The PYP consists of three components: improving pain knowledge and management through a handout, instruction on recording pain in a diary, and behavioral stimulation.	Home visits each lasting 1–1.5 h. Second home visit took place in week 3. The third and final home visit took place at week 6.	8 weeks	Pain intensity	BPI	l baseline (T0), week 4 (T1) and week 8 (T2)	There was no difference in pain scores at T1 (week 4) and T2 (week 8) between the intervention and control groups in patients with a baseline pain score of 1 to 3. Significant differences in pain were found between the intervention and control groups at T1 (*p* = 0.00) and T2 (*p* = 0.00) in patients with a baseline score of 7–10.
Yates et al., 2015 [46]	Australia	189	56	92	Two-thirds (66.1%) of the sample were female with a mean age of 56 years. The median time since diagnosis was 3 months (range: 0–331 months).	Standard cancer care	-	97	Mean age 57.	PMI: The intervention used instructional and cognitive-behavioral strategies and included the delivery of general information about pain and its management, training to help patients learn more adaptive ways of communicating pain, and the development of a pain management plan that included strategies to address patient barriers to effective pain management.	The intervention was administered in two sessions. The first session, approx-imately 30 min long, was held in the outpatient department, and the second session, approxi-mately 15 min long, was conducted by telephone a week later.	8 weeks	Pain intensity	BPI	Follow-up assessment was conducted at 1 week (T1) and 8 weeks (T2) following the second intervention session.	t0 = 4.1 (1.8) T1 = 3.9 (1.8) T2 = 3.5 (1.7)

BPI: Brief Pain Inventory; BPI-T: Brief Pain Inventory—Short Form (Taiwanese version); PMI: Pain Management Index; MEMS: Medication Event Monitoring System (version 6); PKQ-DLV: Pain Knowledge Questionnaire (Dutch version); NRS: Numeric Rating Score 0–10; MPQ –DLV: McGill Pain Questionnaire for the assessment of cancer pain; MPQ-DLV: McGill Pain Questionnaire (Dutch version); EORTC QLC-C30(+3): European Organization for Research and Treatment of Cancer Core Quality of Life Questionnaire; PCL-L: Pain Cognition List for Cancer Patients; APMI: Amsterdam Pain Management Index; POAB–CA: Pain Opioid Analgesics Beliefs Scale—Cancer; CSQ–Cat: Coping Strategies Questionnaire—Catastrophizing; BQ-r: Barrier Questionnaire–revised; KPS: Karnofsky Performance Status; VAS: Visual Analogue Scale; PCS: Pain Catastrophizing Scale; PEP: pain education program;SF-36: Short Form-36 Health Status Surve; PDI: Pain Disability Index; CSI: Central Sensitization Inventory; PROMIS-PF-SF: physical functioning short form; DASS-21: Depression Anxiety Stress scales 21; BTMS: Bodily Threat Monitoring Scale; MQoL: McGill of Life Questionnaire; PSEQ: Pain self-efficacy questionnaire.

## References

[1] Bray F., Laversanne M., Weiderpass E., Soerjomataram I. (2021). The ever-increasing importance of cancer as a leading cause of premature death worldwide. Cancer.

[2] WCRF International Worldwide Cancer Data|World Cancer Research Fund International. https://www.wcrf.org/cancer-trends/worldwide-cancer-data/.

[3] Cancer Research UK. Cancer Statistics for the UK. https://www.cancerresearchuk.org/health-professional/cancer-statistics-for-the-uk.

[4] Ventafridda V., Tamburini M., Caraceni A., De Conno F., Naldi F. (1987). A validation study of the WHO method for cancer pain relief. Cancer.

[5] WCRF International Cancer Survival Statistics. World Cancer Research Fund International. https://www.wcrf.org/cancer-trends/cancer-survival-statistics/.

[6] Santucci C., Carioli G., Bertuccio P., Malvezzi M., Pastorino U., Boffetta P., Negri E., Bosetti C., La Vecchia C. (2020). Progress in cancer mortality, incidence, and survival: A global overview. Eur. J. Cancer Prev..

[7] Cancer Research UK. Breast Cancer Statistics. https://www.cancerresearchuk.org/health-professional/cancer-statistics/statistics-bycancer-type/breast-cancer.

[8] Luo Y.H., Luo L., Wampfler J.A., Wang Y., Liu D., Chen Y.M., Adjei A.A., Midthun D.E., Yang P. (2019). 5-year overall survival in patients with lung cancer eligible or ineligible for screening according to US Preventive Services Task Force criteria: A prospective, observational cohort study. Lancet Oncol..

[9] Ferlay J., Colombet M., Soerjomataram I., Mathers C., Parkin D.M., Pineros M., Bray F. (2019). Estimating the global cancer incidence and mortality in 2018: GLOBOCAN sources and methods. Int. J. Cancer.

[10] Amador M., Matias-Guiu X., Sancho-Pardo G., Contreras Martinez J., de la Torre-Montero J.C., Peñuelas Saiz A., Garrido P., García-Sanz R., Rodríguez-Lescure Á., Paz-Ares L. (2021). Impact of the COVID-19 pandemic on the care of cancer patients in Spain. ESMO Open.

[11] The Effects of Cancer Delays during the COVID-19 Pandemic—Collateral Global. (7 October 2021). Collateral Global. https://collateralglobal.org/article/effects-of-cancer-delays-during-the-covid-19-pandemic.

[12] Singh N., Baby D., Rajguru J.P., Patil P.B., Thakkannavar S.S., Pujari V.B. (2019). Inflammation and cancer. Ann. Afr. Med..

[13] Clohisy D.R., Mantyh P.W. (2003). Bone cancer pain. Clin. Orthop. Relat. Res..

[14] Van den Beuken-van Everdingen M.H.J., de Rijke J.M., Kessels A.G., Schouten H.C., van Kleef M., Patijn J. (2007). High prevalence of pain in patients with cancer in a large population-based study in The Netherlands. Pain.

[15] van den Beuken-van Everdingen M.H.J., de Rijke J.M., Kessels A.G., Schouten H.C., van Kleef M., Patijn J. (2007). Prevalence of pain in patients with cancer: A systematic review of the past 40 years. Ann. Oncol..

[16] Taber J.M., Leyva B., Persoskie A. (2015). Why do people avoid medical care? A qualitative study using national data. J. Gen. Intern. Med..

[17] Neufeld N.J., Elnahal S.M., Alvarez R.H. (2017). Cancer pain: A review of epidemiology, clinical quality and value impact. Future Oncol..

[18] Smith T.J., Staats P.S., Deer T., Stearns L.J., Rauck R.L., Boortz-Marx R.L., Buchser E., Català E., Bryce D.A., Coyne P.J. (2002). Implantable Drug Delivery Systems Study Group. Randomized clinical trial of an implantable drug delivery system compared with comprehensive medical management for refractory cancer pain: Impact on pain, drug-related toxicity, and survival. J. Clin. Oncol..

[19] Kurita G.P., Sjøgren P. (2015). Pain management in cancer survivorship. Acta Oncol..

[20] Pidgeon T., Johnson C.E., Currow D., Yates P., Banfield M., Lester L., Allingham S.F., Bird S., Eagar K. (2016). A survey of patients’ experience of pain and other symptoms while receiving care from palliative care services. BMJ Support. Palliat. Care.

[21] Concepción Pérez Hernández D., Babarro A.A., Aguerri A.R., Villegas Estévez F., Virizuela Echaburu J.A. Guía Para el Abordaje Interdisciplinar del Dolor Oncológico. https://seom.org/seomcms/images/stories/recursos/Guia_GADO_dolor_oncologico.pdf.

[22] World Health Organization (1986). Cancer Pain Relief. https://apps.who.int/iris/handle/10665/43944.

[23] Meuser T., Pietruck C., Radbruch L., Stute P., Lehmann K.A., Grond S. (2001). Symptoms during cancer pain treatment following WHO-guidelines: A longitudinal follow-up study of symptom prevalence, severity and etiology. Pain.

[24] Jara C., Del Barco S., Grávalos C., Hoyos S., Hernández B., Muñoz M., Quintanar T., Meana J.A., Rodriguez C., de Las Peñas R. (2018). SEOM clinical guideline for treatment of cancer pain (2017). Clin. Transl. Oncol..

[25] DiPiro J.T., Talbert R.L., Yee G.C., Wells B.G., Posey L.M. (2014). Pharmacotherapy: A Pathophysiologic Approach 9/E.

[26] Crawford C., Boyd C., Paat C.F., Price A., Xenakis L., Yang E., Zhang W., Evidence for Massage Therapy (EMT) Working Group (2016). The Impact of Massage Therapy on Function in Pain Populations—A Systematic Review and Meta-Analysis of Randomized Controlled Trials: Part I, Patients Experiencing Pain in the General Population. Pain Med..

[27] Nijs J., Wijma A.J., Leysen L., Pas R., Willaert W., Hoelen W., Ickmans K., Van Wilgen C.P. (2019). Explaining pain following cancer: A practical guide for clinicians. Braz. J. Phys. Ther..

[28] Petticrew M., Anderson L., Elder R., Grimshaw J., Hopkins D., Hahn R., Krause L., Kristjansson E., Mercer S., Sipe T. (2013). Complex interventions and their implications for systematic reviews: A pragmatic approach. J. Clin. Epidemiol..

[29] Lee Y.J., Hyun M.K., Jung Y.J., Kang M.J., Keam B., Go S.J. (2014). Effectiveness of education interventions for the management of cancer pain: A systematic review. Asian Pac. J. Cancer Prev. APJCP.

[30] Marie N., Luckett T., Davidson P.M., Lovell M., Lal S. (2013). Optimal patient education for cancer pain: A systematic review and theory-based meta-analysis. Support. Care Cancer.

[31] Champarnaud M., Villars H., Girard P., Brechemier D., Balardy L., Nourhashemi F. (2020). Effectiveness of Therapeutic Patient Education Interventions for Older Adults with Cancer: A Systematic Review. J. Nutr. Health Aging.

[32] Higgins J.P., Altman D.G., Gøtzsche P.C., Jüni P., Moher D., Oxman A.D., Savović J., Schulz K.F., Weeks L., Sterne J.A.C. (2011). The Cochrane Collaboration’s tool for assessing risk of bias in randomised trials. BMJ.

[33] Cashin A.G., McAuley J.H. (2020). Clinimetrics: Physiotherapy Evidence Database (PEDro) Scale. J. Physiother..

[34] De Morton N.A. (2009). The PEDro scale is a valid measure of the methodological quality of clinical trials: A demographic study. Aust. J. Physiother..

[35] De Wit R., Van Dam F.S., Zandbelt L.C., Van Buuren A., Van Der Heijden K., Leenhouts G., Loonstra S. (1997). A Pain Education Program for chronic cancer pain patients: Follow-up results from a randomized controlled trial. Pain.

[36] De Wit R., Van Dam F.S., Loonstra S., Zandbelt L.C., Van Buuren A., Van Der Heijden K., Leenhouts G., Duivenvoorden H.J., Abu-Saad H.H. (2001). Improving the quality of pain treatment by a tailored pain education programme for cancer patients in chronic pain. Eur. J. Pain.

[37] Oldenmenger W.H., Van Der Rijt C.C. (2016). Feasibility of assessing patients’ acceptable pain in a randomized controlled trial on a patient pain education program. Palliat. Med..

[38] Oldenmenger W.H., Smitt P.A.E.S., Van Montfort C., De Raaf P.J., Van Der Rijt C.C. (2011). A combined pain consultation and pain education program decreases average and current pain and decreases interference in daily life by pain in oncology outpatients: A randomized controlled trial. Pain.

[39] Yildirim Y., Cicek F., Uyar M. (2009). Effects of Pain Education Program on Pain Intensity, Pain Treatment Satisfaction, and Barriers in Turkish Cancer Patients. Pain Manag. Nurs..

[40] Lai Y.H., Guo S., Keefe F.J., Tsai S., Chien C., Sung Y., Chen M. (2004). Effects of brief pain education on hospitalized cancer patients with moderate to severe pain. Support. Care Cancer.

[41] Pas R., Leysen L., De Goeij W., Vossebeld L., Van Wilgen P., De Groef A., De Kooning M. (2020). Pain Neuroscience Education in cancer survivors with persistent pain: A pilot study. J. Bodyw. Mov. Ther..

[42] De Groef A., Evenepoel M., Van Dijck S., Dams L., Haenen V., Wiles L., Catley M., Vogelzang A.A., Olver I., Hibbert P. (2023). Feasibility and pilot testing of a personalized eHealth intervention for pain science education and self-management for breast cancer survivors with persistent pain: A mixed-method study. Support. Care Cancer.

[43] Kim H.J., Shin S.J., Kim S.H., An S., Rha S.Y., Ahn J.B., Cho B.C., Choi H.J., Sohn J., Rha S. (2013). Randomized controlled trial of standardized education and telemonitoring for pain in outpatients with advanced solid tumors. Support. Care Cancer.

[44] Wells N., Hepworth J.T., Murphy B.A., Wujcik D., Johnson R. (2003). Improving Cancer Pain Management Through Patient and Family Education. J. Pain Symptom Manag..

[45] Van Der Peet E.H., Van Den Beuken-Van Everdingen M.H.J., Patijn J., Schouten H.C., Van Kleef M., Courtens A.M. (2009). Randomized clinical trial of an intensive nursing-based pain education program for cancer outpatients suffering from pain. Support. Care Cancer.

[46] Yates P., Edwards H., Nash R., Aranda S., Purdie D.M., Najman J.M., Skerman H., Walsh A.M. (2004). A randomized controlled trial of a nurse-administered educational intervention for improving cancer pain management in ambulatory settings. Patient Educ. Couns..

[47] Lovell M.R., Luckett T., Boyle F.M., Phillips J., Agar M., Davidson P.M. (2014). Patient education, coaching, and self-management for cancer pain. J. Clin. Oncol..

[48] Oldenmenger W.H., Geerling J.I., Mostovaya I., Vissers K.C.P., de Graeff A., Reyners A.K.L., Van der Linden Y.M. (2018). A systematic review of the effectiveness of patient-based educational interventions to improve cancer-related pain. Cancer Treat. Rev..

[49] Prevost V., Delorme C., Grach M., Chvetzoff G., Hureau M. (2016). Therapeutic Education in Improving Cancer Pain Management. Am. J. Hosp. Palliat. Med..

[50] Bennett M.I., Bagnall A.M., Closs J.S. (2009). How effective are patient-based educational interventions in the management of cancer pain? Systematic review and metaanalysis. Pain.

[51] Lepri B., Romani D., Storari L., Barbari V. (2023). Effectiveness of Pain Neuroscience Education in Patients with Chronic Musculoskeletal Pain and Central Sensitization: A Systematic Review. Int. J. Environ. Res. Public Health.

[52] Van Ittersum M., Van Wilgen C.P., Van Der Schans C.P., Lambrecht L., Groothoff J.W., Nijs J. (2014). Written Pain Neuroscience Education in Fibromyalgia: A Multicenter Randomized Controlled Trial. Pain Pract..

[53] Louw A., Diener I., Butler D., Puentedura E.J. (2011). The Effect of Neuroscience Education on Pain, Disability, Anxiety, and Stress in Chronic Musculoskeletal Pain. Phys. Med. Rehabil..

[54] Martin M.Y., Pisu M., Kvale E.A., Johns S.A. (2012). Developing effective cancer pain education programs. Curr. Pain Headache Rep..

[55] Mosadeghrad A.M. (2014). Factors influencing healthcare service quality. Int. J. Health Policy Manag..

[56] Nijs J., Van Wilgen C.P., Van Oosterwijck J., Van Ittersum M., Meeus M. (2011). How to explain central sensitization to patients with ‘unexplained’ chronic musculoskeletal pain: Practice guidelines. Man. Ther..

[57] Caraceni A., Hanks G., Kaasa S., Bennett M.I., Brunelli C., Cherny N., Zeppetella G. (2012). Use of opioid analgesics in the treatment of cancer pain: Evidence-based recommendations from the EAPC. Lancet Oncol..

[58] Fernández-Lao C., Cantarero-Villanueva I., Fernández-De-Las-Peñas C., Del-Moral-Ávila R., Menjón-Beltrán S., Arroyo-Morales M. (2011). Widespread Mechanical Pain 46 Hypersensitivity as a Sign of Central Sensitization after Breast Cancer Surgery: Comparison between Mastectomy and Lumpectomy. Pain Med..

[59] Liu S., Liu Y.W., Yue D., Liu G. (2014). Protease-activated receptor 2 in dorsal root ganglion contributes to peripheral sensitization of bone cancer pain. Eur. J. Pain.

